# Electrical Impedance Tomography in Congenital Diaphragmatic Hernia

**DOI:** 10.6061/clinics/2021/e3210

**Published:** 2021-07-08

**Authors:** Rafael Gonçalves Comparini, Mario Cicero Falcão, Cíntia Johnston, Werther Brunow de Carvalho

**Affiliations:** Instituto da Crianca e do Adolescente, Hospital das Clinicas HCFMUSP, Faculdade de Medicina, Universidade de Sao Paulo, Sao Paulo, SP, BR.

Congenital diaphragmatic hernia (CDH) is a severe defect with an estimated incidence of 1:3000 live births (2). This anomalous condition is characterized by an absence of separation between the thoracic and abdominal cavities during fetal development. Some of the first hypotheses about this congenital defect postulated that the presence of abdominal viscera in the thoracic cavity caused pulmonary hypoplasia by pushing the abdominal contents into the developing lungs (1,2).

After several studies in animal models, a new theory was presented to explain pulmonary hypoplasia in CDH. According to this theory, the initial lesion occurs in the early stages of fetal development and organogenesis, which promotes hypoplasia of both lungs and ipsilateral lung compression by herniation of the abdominal contents. This prevents the branching of bronchi, bronchioles, and pulmonary arteries and veins, resulting in hypoplasia of the pulmonary acini. The terminal bronchioles are reduced and the alveolar septa are thickened, which results in relatively immature lungs and vascular hypoplasia. This theory explains the variability of pulmonary hypoplasia in patients with CDH (3).

In addition to the underdeveloped lungs, CDH also involves an abnormal development of pulmonary structures, mainly vessels and bronchi. This abnormal development increases vascular resistance and culminates in significant pulmonary hypertension, which is the main cause of morbimortality in children with CDH (1,2).

In patients with CDH, the total number of lung vascular beds, arteries, and veins per lung unit are reduced, and vascular reshaping and hyperplasia in the tunica media of small arteries may occur. The scarcity of arteries and veins and reshaping of the lung vessels contribute to the unresponsive pulmonary hypertension, which in turn leads to refractory shock and death. The explanation for these events is the reactivity of the altered vessels due to an imbalance of autonomous innervation, vasoconstrictor mediators, and vasodilators. After birth, a combination of right ventricular hypertrophy, left ventricular hypoplasia, and an extremely altered lung architecture results in severe pulmonary hypertension, which is often unresponsive to treatment (3-5).

Electrical impedance tomography (EIT) is a new, noninvasive, radiation-free method that can be used to assess pulmonary physiology during mechanical ventilation. EIT is a bedside method that uses high-frequency and low-amplitude electrical currents to obtain cross section images of both lungs (6,7).

The development of EIT for use in newborns has emerged as a noninvasive method of continuous ventilation monitoring, because it generates functional images that allow continuous evaluation of pulmonary ventilation in different lung regions and lung perfusion in real time (6,8). This technique administers high-frequency and low-amplitude electrical currents through 16 electrodes arranged on the chest in order to acquire axial pulmonary images (7). The electrical currents are transmitted through the thorax and follow trajectories that vary according to the electrical resistance of the chest wall and lung tissue, which can allow or hinder their progression.

The electrical potentials obtained on the chest are gauged and used to determine the distribution of intrathoracic electrical impedance, and images are created to represent the air distribution area in the lungs (corresponding to areas of greater resistance to the passage of the electric current). Thus, EIT explores the differences in electrical impedance generated by the replacement of tissue conductivity obtained from the range in the tidal volume of gases during breathing (8).

A 3-hour old infant with a non-corrected left CDH was referred for EIT for the observation of pulmonary hypoplasia. As shown in Figure 1, the distribution of pulmonary ventilation was lower on the side of the diaphragmatic defect than on the side with the normal lung (98% *versus* 2%). The distribution pattern showed compensatory ventilation in the right lung. A slight difference was observed between gravity- and non-gravity-dependent areas (56% *versus* 44%). Plethysmography showed an inferior tidal volume distribution on the compromised side (Figure 1).

## Figures and Tables

**Figure 1:**
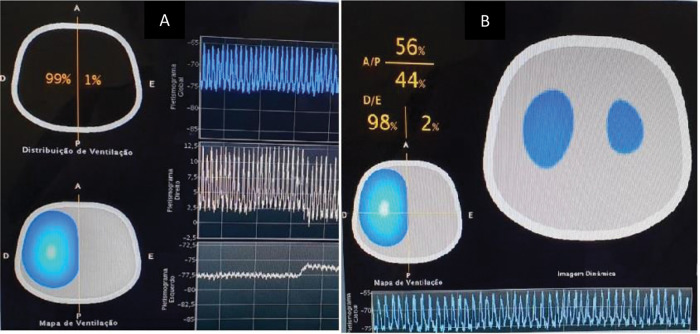
**A.** Ventilation distribution in the right (R) and left (L) half thorax. The white image represents the area with an increased aeration distribution, and the blue image shows decreasing aeration distribution. Graphics represent plethysmogram of tidal volume per region: global (superior), right (middle), and left (inferior). **B.** Ventilation distribution over cross sections: Anterior/Posterior and R/L (images obtained from screen equipment).
